# The Nasal Microbiome in ANCA-Associated Vasculitis: Picking the Nose for Clues on Disease Pathogenesis

**DOI:** 10.1007/s11926-021-01015-9

**Published:** 2021-07-01

**Authors:** G. J. Dekkema, A. Rutgers, J. S. Sanders, C. A. Stegeman, P. Heeringa

**Affiliations:** 1 Department of Rheumatology and Clinical Immunology, University Medical Center Groningen, University of Groningen, Groningen, The Netherlands; 2 Division of Nephrology, Department of Internal Medicine, University Medical Center Groningen, University of Groningen, Groningen, The Netherlands; 3 Department of Pathology and Medical Biology, University Medical Center Groningen, University of Groningen, Hanzeplein 1 EA11, 9713 GZ Groningen, The Netherlands

**Keywords:** Microbiome, ANCA, Vasculitis, *Staphylococcus aureus*, Autoimmune disease

## Abstract

**Purpose of Review:**

The onset and progression of small vessel vasculitis associated with anti-neutrophil cytoplasmic antibodies has been linked to microbial infections. Here, we provide a brief overview of the association of nasal colonization of *Staphylococcus aureus* with ANCA-associated vasculitis (AAV) and discuss several recent studies mapping the nasal microbiome in AAV patients in particular.

**Recent Findings:**

Nasal microbiome studies revealed dysbiosis as a common trait in active AAV which tends to normalize upon immunosuppressive treatment and quiescent disease. However, due to differences in study design, patient selection, and methodology, the reported microbiome profiles differ considerably precluding conclusions on causal relationships.

**Summary:**

The microbiome is an emerging area of research in AAV warranting further investigation. Ideally, such studies should be combined with mechanistic studies to unravel key elements related to host-microbe interactions and their relevance for AAV pathogenesis.

## Introduction

Throughout life, the human body is constantly exposed to and co-exists with huge numbers of microbes including bacteria, viruses, and fungi. Vast numbers of microbes colonize the epithelial surfaces of the skin and body cavities. Most colonizing microbes are considered commensals which compete for space with pathogenic bacteria and interact with the host shaping the functions of the immune system. Others are true symbionts contributing importantly to body homeostasis and metabolism [1]. Collectively, the communities of commensal, symbiotic, and pathogenic microorganisms on our skin and inhabiting our body’s cavities are referred to as the human microbiome. Over the last decades, technological advances in sequencing and computational methods have provided detailed insights into the diversity of the microbial communities harboring our body cavities and have led to a surge in studies on the human microbiome in relation to health and disease [2]. From these studies, it has become clear that maintaining a diverse microbiome in which beneficial species dominate is essential for health whereas a loss of microbiome diversity, often referred to as dysbiosis, has been linked to various chronic inflammatory disorders including autoimmune diseases [2, 3].

Anti-neutrophil cytoplasmic autoantibody (ANCA)–associated vasculitides (AAV) are rare, severe autoimmune diseases characterized by necrotizing inflammation of small- to medium-sized blood vessels and the presence of ANCA [4••]. Based on clinical and pathological features, three disease subgroups can be distinguished: granulomatosis with polyangiitis (GPA), microscopic polyangiitis (MPA), and eosinophilic GPA (EGPA). ANCA in AAV are directed against the neutrophil lysosomal proteins, proteinase 3 (PR3-ANCA) or myeloperoxidase (MPO-ANCA). Although PR3-ANCA and MPO-ANCA can be found across the AAV spectrum, PR3-ANCA is typically associated with GPA whereas MPA patients usually present with MPO-ANCA [4••]. More recently, it has been proposed that it may be more appropriate to distinguish patients based on ANCA specificity as whole genome association studies indicated that PR3-ANCA and MPO-ANCA patients are genetically distinct and may have a different pathogenesis and clinical course [5, 6].

Clinical manifestations of GPA include necrotizing granulomatous inflammation of the upper and lower airways and glomerulonephritis. In GPA, the majority of patients initially present with sinonasal involvement presenting as nasal obstruction and rhinitis, which in more than 60% of patients persists during disease remission [7]. In contrast to GPA, patients with MPA often present with non-granulomatous glomerulonephritis and pulmonary involvement.

Culture-based methods have demonstrated an increased frequency of nasal carriage of *S. aureus* in GPA patients (72%) compared to healthy individuals (28%) and patients with chronic rhinosinusitis (25%) which has been linked to an increased risk for disease relapse [8, 9]. Here, we will provide a brief overview of the current literature pertaining to the role of *S. aureus* in the etiopathogenesis of AAV focusing primarily on GPA. Subsequently, we will review several recent studies leveraging novel high-throughput sequencing methods for the in-depth characterization of the nasal microbiome in GPA and discuss their implications for our understanding of the host-microbe interactions in GPA development.

## The Link Between *S. aureus* and AAV

The etiology of AAV is unknown but is generally considered to be the result of a complex interplay between genetic background, environmental factors, including microbes, and possibly age-related changes in innate and adaptive immunity. Since its first description, and subsequently supported by clinical and experimental observations, the development of GPA has been linked to infections [4••]. Several infectious agents have been implicated in the pathogenesis of AAV with most attention being given to *S. aureus* [10]. As mentioned before, chronic nasal carriage of *S. aureus* is more frequent in GPA patients compared to healthy controls [8, 9]. Interestingly, nasal *S. aureus* carriage in GPA has been found to correlate with endoscopically proven endonasal disease activity and a higher relapse rate. In addition, treatment for 24 months with the antibiotic co-trimoxazole was shown to prevent disease relapses [8, 11]. Post hoc analysis of two randomized clinical trials confirmed the association with chronic *S. aureus* carriage and increased relapse rate [12]. However, this study also found that relapses in GPA patients do occur in the absence of *S. aureus* positivity as well as upon prophylactic antibiotic treatment. This led the authors to suggest that the increased relapse risk is not primarily due to *S. aureus* carriership but may relate to other, yet unknown, genetically determined or environmental factors [12]. Important to note is that recent data from a prospective observational cohort study from France confirmed the increased frequency of *S. aureus* nasal colonization in AAV patients but found no association of nasal carriership of this bacteria or co-trimoxazole treatment in prophylactic dosages with relapse rates [13].

In contrast to GPA, data on *S. aureus* carriage in MPA and EGPA data is scarce. Only one study investigated *S. aureus* carriage in MPA and did not observe a correlation between disease relapse and *S. aureus* carriage [12]. Moreover, in the study by Tan and colleagues from France, rates of *S. aureus* carriage did not differ between AAV subgroups, i.e. GPA, MPA, and EGPA, nor between PR3-ANCA- and MPO-ANCA-positive AAV patients [13].

To determine whether specific *S. aureus* types are associated with AAV pathogenesis, our group performed a more detailed retrospective genetic analysis on stored *S. aureus* isolates from AAV patients [14, 15]. This analysis demonstrated that PR3-ANCA and MPO-ANCA patients carry highly diverse *S. aureus* types similar to those found in the general population. Importantly, in sera from both PR3-ANCA- and MPO-ANCA-positive patients, IgG antibody responses against 59 *S*. *aureus* antigens were lower compared to those from HC. This effect was independent of treatment suggesting that AAV patients are less capable of initiating a protective humoral immune response to *S. aureus* [14].

**Fig. 1:**
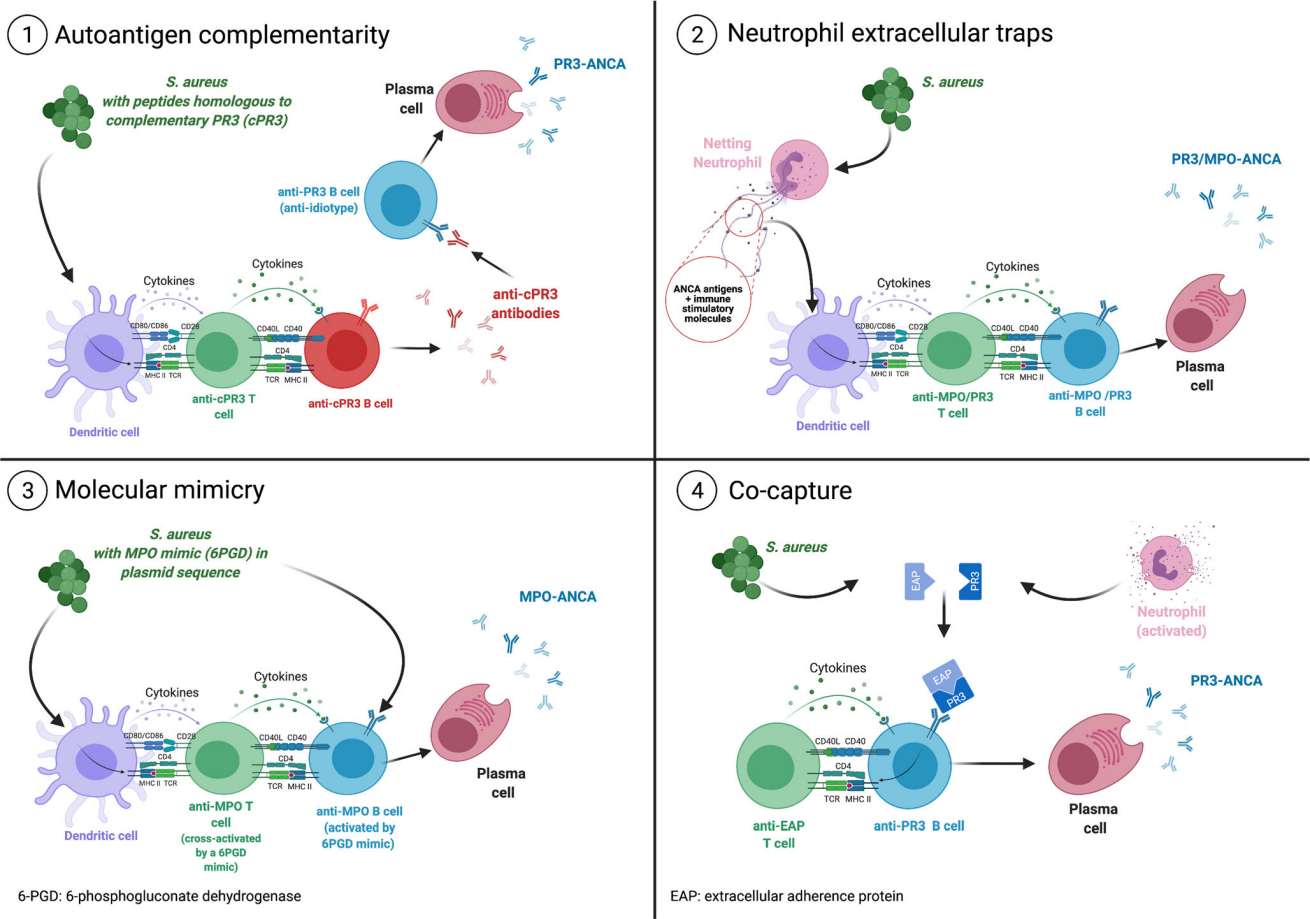
Graphical presentation of four putative mechanisms proposed implicating *S. aureus* in the breach of tolerance towards the ANCA antigens, PR3 and MPO. See text for explanation. Created with BioRender.com

Although the exact role of *S. aureus* in disease pathogenesis is far from clear, it has been postulated that *S. aureus* may induce a break in tolerance to PR3 and MPO in predisposed individuals by one or more of the following mechanisms (Fig. 1).

First, Pendergraft and colleagues reported that *S. aureus* harbors proteins that are highly homologous to a complementary form of human PR3 (cPR3) [16]. cPR3 is the protein translated from the antisense DNA strand of the PR3 gene. Immunization of mice with human cPR3 or a synthetic homolog resulted in the production of antibodies directed against cPR3 and, via idiotype-anti-idiotype responses, ANCA directed against PR3, indicating that these homologous proteins can contribute to tolerance breakdown. However, the relevance of this finding remains controversial since increased reactivity against cPR3 could not be confirmed in an independent AAV cohort [17].

Second, *S. aureus* are potent inducers of neutrophil extracellular traps (NETs), extracellular complexes of DNA and antimicrobial factors secreted by neutrophils as part of their antimicrobial defense mechanism to limit bacterial spreading [18]. The antimicrobial factors associated with NETs include, among others, the ANCA antigens MPO and PR3 and alarmins such as LL37 and the non-histone nuclear protein HMGB1 [19, 20]. The latter can act as adjuvants to stimulate immune responses rendering NETs potentially highly immunogenic. Thus, NETs may initiate or maintain the autoimmune response in AAV by exposing the ANCA antigens to the immune system while at the same time providing immune-stimulatory effects. Indeed, studies in mice have demonstrated the induction of ANCA upon injection of activated myeloid dendritic cells loaded with NET components [19].

As a third mechanism, molecular mimicry, a mechanism based on structural antigenic similarities between infectious agents and self-proteins, has been proposed. In a recent study, Ooi and colleagues identified a *S. aureus* plasmid–encoded peptide, 6-phosphogluconate dehydrogenase (6PGD)391–410, that was homologous to a previously characterized immunodominant MPO T cell epitope [21•]. The 6PGD protein and peptide were found to be immunogenic in humans as evidenced by detectable anti-6PGD IgG levels in sera from healthy controls and AAV patients. Moreover, in mice, immunization with the 6PGD391–410 peptide or with *S. aureus* containing a plasmid expressing 6PGD391–410, induced anti-MPO autoimmunity. In these mice, injection of a low dose of heterologous anti-glomerular basement membrane globulins triggered glomerular MPO deposition and ensuing glomerulonephritis. Overall, these studies suggest that molecular mimicry may mechanistically contribute to a breach in tolerance towards MPO. However, since immunoreactivity towards 6PGD391–410 was found in healthy controls as well, it certainly is not the only factor involved [21•].

A fourth hypothetical yet intriguing mechanism described recently, here referred to as co-capture, implicates the *S. aureus*–derived extracellular adherence proteins (EAP) and staphylococcal peroxidase inhibitor (SPIN) in the induction of pathogenic ANCA [22]. EAP and SPIN are enzyme inhibitors produced by *S. aureus* that target and form complexes with PR3 and MPO, respectively [23, 24]. Importantly, natural low-affinity antibodies directed against PR3 and MPO (natural ANCAs) have been demonstrated in sera of healthy controls (HCs) indicative of the presence of natural ANCA-producing B cells [25]. Hypothetically, these EAP:PR3 or SPIN:MPO complexes may be recognized by those natural ANCA-producing B cells and become internalized. Since B cells are professional antigen-presenting cells, processing of these complexes followed by presentation of bacterial peptides by MHC-II molecules may activate EAP- or SPIN-specific T helper (Th) cells. In turn, these EAP- or SPIN-specific Th cells provide help to the natural ANCA-producing B cells, allowing isotype switching and affinity maturation leading to the production of high-affinity pathogenic IgG ANCA-initiating autoimmune disease.

Of these four hypotheses, the formation of NETs might offer the most elegant and plausible explanation for the role of *S. aureus* in the induction of auto-immunity in AAV. However, direct evidence to support *S. aureus*–mediated NETosis in the immunogenesis of AAV is still lacking.

Using similar mechanisms as described above, *S. aureus* may also play a role in provoking relapses. EAP:PR3 or SPIN:MPO complexes could reactivate PR3- or MPO-specific B cells. In addition, *S. aureus*–derived constituents such as CpG motifs and superantigens may also reactivate the autoinflammatory response in AAV patients in an antigen-independent way. This notion is supported by studies showing that stimulation of peripheral blood mononuclear cells (PBMCs) derived from GPA patients with either CpG and IL-2 or CpG, IL-21, and BAFF triggers PR3-ANCA production in vitro [26–28]. In addition, *S. aureus*–derived superantigens such as TSST-1 may activate autoreactive immune cells by forcing interaction between T and B cells or T cells and antigen-presenting cells [29].

More recently, Krebs and colleagues proposed a role for pathogen-induced tissue-resident memory (TRM) CD4+ T cells displaying Th17 features, termed TRM17 cells, in the exacerbation of renal pathology in AAV [30••]. Analysis of kidney tissue of patients with ANCA-associated glomerulonephritis revealed the presence of relatively large numbers of TRM17 cells. Interestingly, these authors further demonstrated that infection of mice with *S. aureus* induced TRM17 cells that persisted in kidney tissue. Moreover, induction of crescentic glomerulonephritis in *S. aureus*–infected mice promoted renal IL-17A production and worsened disease manifestations suggesting a pathogenic role of pathogen-induced TRM17 cells [30••].

Finally, a retrospective analysis comparing *S. aureus* isolates from MPO-ANCA and PR3-ANCA patients suggests that differences in expression of virulence genes may contribute to disease pathogenesis. In that study, genetic loci for several leukocidins and hemolysins were associated with PR3-ANCA isolates [15]. Leukocidins are pore-forming cytotoxins that target immune cells and incite a form of cell death termed leukotoxic hypercitrullination (LTH) by inducing Ca2+ ion influx and osmotic lysis [31]. Like NETosis, LTH causes extrusion of chromatin-like structures and release of granule proteins and thereby may promote the local inflammatory response.

In summary, *S. aureus* may contribute to tolerance breakdown as well as trigger inflammatory responses leading to AAV in susceptible subjects in various, non-mutually exclusive, ways. However, direct evidence implicating *S. aureus* as the instigator of autoimmunity or driver of the inflammatory response in AAV is lacking. Hence, it cannot be ruled out that other infectious agents contribute to AAV pathogenesis. Indeed, the occurrence of ANCAs has been reported in several infectious conditions including viral (Ross River virus, hepatitis B and C, Epstein-Barr virus, or parvo B-19), bacterial (*Streptococcus*), fungal, and parasitic infections [32]. However, whether these elicited ANCAs are pathogenic is still under debate and most likely depends on their specificity for pathogenic epitopes [33]. Nevertheless, microbial agents other than *S. aureus* cannot be excluded as potential triggers or amplifiers in AAV pathogenesis. In addition, it should be taken into account that up until recently most studies in AAV investigating the nasal microbial composition were not designed to comprehensively profile microbial diversity but relied on culture-based methods.

## *S. aureus* and Beyond: the Nasal Microbiome in GPA

Given that the vast majority of GPA patients present with a history of sinonasal inflammation and that the nasal cavities serve as a major reservoir for opportunistic pathogens, it is not surprising that there has been an increased interest to map the nasal microbiome of GPA patients (Table 1).

**Table 1:** Overview of microbiome studies in ANCA vasculitis

	Study techniques	Cohort	Immunosuppressive treatment	Sample	Main results
Rhee et al. [34••]	16S rRNA (V1-V2) and ITS 1 region sequencing	GPA = 60 (PR3+ 60%, MPO+ 25%) HC = 41 Of which: Active disease = 25% Remission = 25% Positive VDI = 47%	Glucocorticoids: Current 42%, past 52% Non-glucocorticoid: Curent 53%, past 70% Antibiotics Current 25%, past 48% Past is within 6 months	Nasal, middle meatus	- GPA significantly lower relative diversity of microbiome - Lower abundance of *P. acnes* and *S. epidermidis* in GPA - Disease activity associated with lower abundance of *Malasseziales* - Non-glucocorticoid immunosuppressive treatment associated with normalization of microbiome
Wagner et al. [35]	16S rRNA shotgun sequencing SEED profiling *Staphylococcus aureus* culture and whole genome sequencing	GPA = 66 ANCA: not specified HC = 11 DC (MPA + EGPA) = 13 Of which: Active disease = 33.3% Remission = 66.6% Follow-up swaps of all active patients	All immunosuppressive treatment within 1 year before sampling: Active GPA: 75% Remission: 47.6% Disease control: 69.2% Healthy controls: 0% Glucocorticoids: Active GPA: 75% Remission: 46.7% Disease control: 69.2 %	Nasal cavity, both sides	- GPA significantly lower relative diversity of microbiome - Shotgun seq: different composition *Staphylococcus* sp. Active GPA patients had higher abundance of *S. aureus*. Healthy controls had higher abundance of *S. epidermidis* - Functional analysis (SEED): most significant associations between groups with chorismate and B12 pathway - Significantly higher *S. aureus* positive cultures in active GPA patients (66.7%) vs remission (34.1%) and healthy controls (18.2%) - Higher abundance of *S. aureus* in remission GPA was not associated with increased relapse risk
Lamprecht et al. [36]	16S rRNA (V3-V4) next-generation sequencing	GPA = 29 (PR3+ 83%, MPO+ 3%) RA = 21 HC = 27 Of which: Active disease = 21% Remission = 79% All patients had (history) of ENT involvement	Glucocorticoids: Current: 97% Non-glucocorticoid: Current: 100% No current antibiotics within 3 weeks before sampling	Nasal cavity, both sides	- Trend towards lower relative diversity in GPA - Increase in bacteria assigned to family of *Streptococcaceae*, *Pasteurellaceae*, and *Prevortellacea*. Decrease in *Corynebacteriaceae*, *Staphylococcaceae*, and *Propionibacteriaceae* in GPA *- Staphylococcaceae* more abundant during remission *- Streptococcaceae* and *Planococcaceae* more abundant in patients with ENT activity, *Corynebacteriaceae* was decreased - Increased detection of *S. aureus* in GPA patients
Rhee et al. [37••]	16S rRNA (V1-V2)	GPA = 19 Of which: Relapse during study = 9 Remission = 10 Longitudinal study, on average 3–4 visits per patient	Future relapse = 67% Glucocorticoid = 33% Remission = 100% Glucocorticoid = 50%	Nasal cavity	*- Corynebacteriaceae* and *Staphylococcaceae* most abundant in nasal microbiome - Increase in *Staphylococcaceae* at visit before relapse - Decrease of *Staphylococcaceae* and increase in *Corynebacteriaceae* during relapse - Higher abundance of *C. tuberculostearium* which correlates to high ANCA titer and relapse
Fukui et al. [38•]	16S rRNA (V4)	MPA = 14, GPA = 2 ANCA: not specified Sarcoidosis = 21 Of which: Active AAV = 16 Active sarcoidosis = 21 All but one patient with sarcoidosis had pulmonary involvement	No immunosuppressive medication at time of sampling	Lung, BALF	- Alpha diversity was inversely correlated to BVAS in AAV - No difference in alpha diversity when analyzing all taxa between AAV and sarcoidosis - Significant difference in alpha diversity when studying oral or non-oral microbiome separately between AAV and sarcoidosis
Najem et al. [39] (abstract)	16S rRNA (V1-V2)	GPA = 49 ANCA: not specified HC 14 Of which: Active disease = 59% Remission = 41%	Not specified	Gut, fecal samples	- Patients with active GPA had significant altered gut microbial composition - No difference between HC and remission - Relative abundance taxa *Dialister* and *Prevotella* significantly different between HC and active GPA *- Dialister* and *Prevotella* were different between active and remission AAV. The relative abundance of *Faecalibacterium* and *Sutterella* was different between active and remission newly diagnosed AAV

*ANCA* anti-neutrophil cytoplasmic antibodies, *GPA* granulomatosis with polyangiitis, *MPA* microscopic polyangiitis, *EGPA* eosinophilic granulomatosis with polyangiitis, *AAV* ANCA-associated vasculitis, *HC* healthy control, *PR3* proteinase 3, *MPO* myeloperoxidase, *ITS* internal transcribed spacer

The first study using culture-independent sequencing methods to comprehensively portray the community of nasal bacteria and fungi in GPA patients employed 16S rRNA and internal transcribed spacer gene sequencing [34••]. The study compared samples taken from 41 healthy volunteers and 60 GPA patients in remission of which the majority had experienced sinonasal involvement at any time during their disease. GPA patients were found to have a significantly altered composition of the nasal microbiome compared to that of healthy controls characterized by a lower abundance of *Propionibacterium acnes* and *Staphylococcus epidermidis* in GPA [34••]. Interestingly, both bacterial strains are known to compete with *S. aureus* for nasal colonization [40, 41]. However, in contrast to the culture-dependent studies described earlier, no difference in the abundance of *S. aureus* was found between GPA patients and healthy participants. At the mycobiome level, Rhee et al. reported a lower abundance of *Malasseziales* in GPA patients with active disease compared to patients in remission and healthy controls [34••]. Arguably the most interesting observation from this study was that differences in microbiome composition appeared to be largely driven by the type of immunosuppressive therapy. The nasal microbiome of patients treated with non-glucocorticoid immunosuppressants was comparable to that of healthy volunteers, an effect not seen in patients receiving glucocorticoids only [34••].

A more recent study by Wagner and colleagues also highlighted the presence of nasal dysbiosis in patients with GPA [35]. Using 16S rRNA shotgun sequencing, they identified an altered composition of the *Staphylococcus* genus, with a higher abundance of *S. aureus* in patients with active GPA and higher prevalence of *S. epidermidis* in healthy volunteers. The composition of the *Staphylococcus* genus did not differ between patients with active disease and those in remission. Samples from MPA and EGPA patients, included as disease controls in this study, did however display a lower prevalence of *S. aureus* suggesting a different role of the *Staphylococcus* genus in the microbiome of EGPA and MPA as compared to GPA. The increased abundance of *S. aureus* in GPA patients was confirmed by a significantly higher frequency of *S. aureus–*positive cultures in patients with active disease (66.7%) when compared to patients in remission (34.1%) and healthy volunteers (18.2%) which is in line with the aforementioned culture-based studies. However, no significant correlation was found between *S. aureus* positivity in patients in remission and the occurrence of future disease relapses [35].

Thirdly, Lamprecht and colleagues also reported changes in nasal microbiome composition in GPA as compared to healthy controls and rheumatoid arthritis patients (RA) [36]. In this study, microbiome diversity tended to be reduced in GPA samples compared to healthy controls and was significantly reduced compared to RA. Moreover, a shift in bacterial families was observed in both GPA and RA samples showing increased abundance of *Planococcaceae* and decreased *Moraxellaceae*, *Tissierellaceae*, *Staphylococcaceae*, and *Propionibacteriaceae*. In GPA samples, a decreased abundance of *Corynebacteriaceae* and *Aerococcaceae* was observed compared to healthy controls. Importantly, closer inspection of the data revealed that bacterial composition was influenced by local disease activity. GPA patients with nasal involvement displayed a lower dominance of *Corynebacteriaceae* whereas the abundance of *Streptococcaeae* and *Planococcaceae* was increased. Finally, detection of *S. aureus* by qPCR revealed a higher frequency of *S. aureus* colonization in GPA compared with RA and healthy control samples corroborating data from culture-based studies [36].

Lastly, a recent study by Rhee and colleagues studied changes in the nasal microbiome in GPA patients during their disease course. Nineteen GPA patients in remission were included and followed longitudinally with a median follow-up of 13.8 months during which time 9 patients relapsed and 10 remained in remission. At baseline, *Staphylococcaceae* and *Corynebacteriaceae* were the most abundant genera and their ratio was stable during remission. Upon relapse, however, this ratio changed significantly. At the genus level, 3 to 4 months prior to relapse, an increase in *Staphylococcaceae* was observed, whereas *Corynebacteriaceae* were more dominant during relapse. Interestingly, an increase in the relative abundance of *Corynebacterium tuberculostearicum* significantly correlated with a higher ANCA titer and disease relapse [37••].

In summary, despite the fact that the microbiome profiles reported are quite heterogeneous, a common theme across the 4 nasal microbiome studies seems to be the altered composition of the *Staphylococcus* genus and its competitors for colonization. One possibility is that the pathogenic effects of *S. aureus* in GPA occur in an immunological context that is not only dependent on bacterial colonization but also requires the expression of certain virulence factors (e.g., leukocidins) and an impaired host response. As mentioned earlier, *S. aureus*–positive GPA patients mount a significantly lower, potentially protective, humoral response against various *S. aureus*–derived secreted and surface proteins. In addition, evidence exists that in GPA patients, the nasal barrier function is disturbed due to reduced epithelial ciliary motility and production of antimicrobial proteins such as LL37 and beta-defensin-3 (hBD-3) [42, 43]. Collectively, these observations support the notion that increased *S. aureus* carriage in conjunction with disturbances in innate and adaptive immune response may contribute to GPA pathogenesis although a direct causal relationship remains to be proven. In addition, more studies are needed to clarify the role of other bacteria, such as *Corynebacteriaceae*, in disease pathogenesis.

One important observation from the study by Rhee and colleagues is that in patients in remission on non-glucocorticoid immunosuppressive treatment, the nasal microbiome tends to normalize [34••]. One explanation for this unexpected finding proposed by the authors is that patients not receiving immunosuppressive therapy still have subclinical disease activity affecting the nasal microbiome, thus suggesting that immunosuppressant therapy may be beneficial in treating nasal dysbiosis. Other possibilities include either a direct influence of glucocorticoids on the composition of the microbiome or an indirect effect due to suppression of the immune response to potential pathogens.

Besides the nasal microbiome, two relatively small studies have been reported that focused on the microbiota of the lungs and gut in AAV, respectively (Table 1). Fukui et al. studied the microbiome of the lungs comparing broncho-alveolar fluid (BALF) samples of 16 AAV and 21 sarcoidosis patients [38•]. Their analysis showed that the diversity of the pulmonary microbiome was inversely correlated to disease activity illustrated by a relatively less rich microbiome in patients with a higher composite disease activity score (Birmingham Vasculitis Activity Score).

In many other auto-immune diseases, gut microbiome dysbiosis has been a major focus of investigations. In AAV, only one study examined the gut microbiome so far [39] suggesting gut dysbiosis in patients with active GPA. In line with the nasal microbiome data, the gut microbiome appears to normalize upon immunosuppressive treatment whereas no differences were found between patients in disease remission and healthy volunteers.

## Conclusion and Future Perspectives

**Fig. 2:**
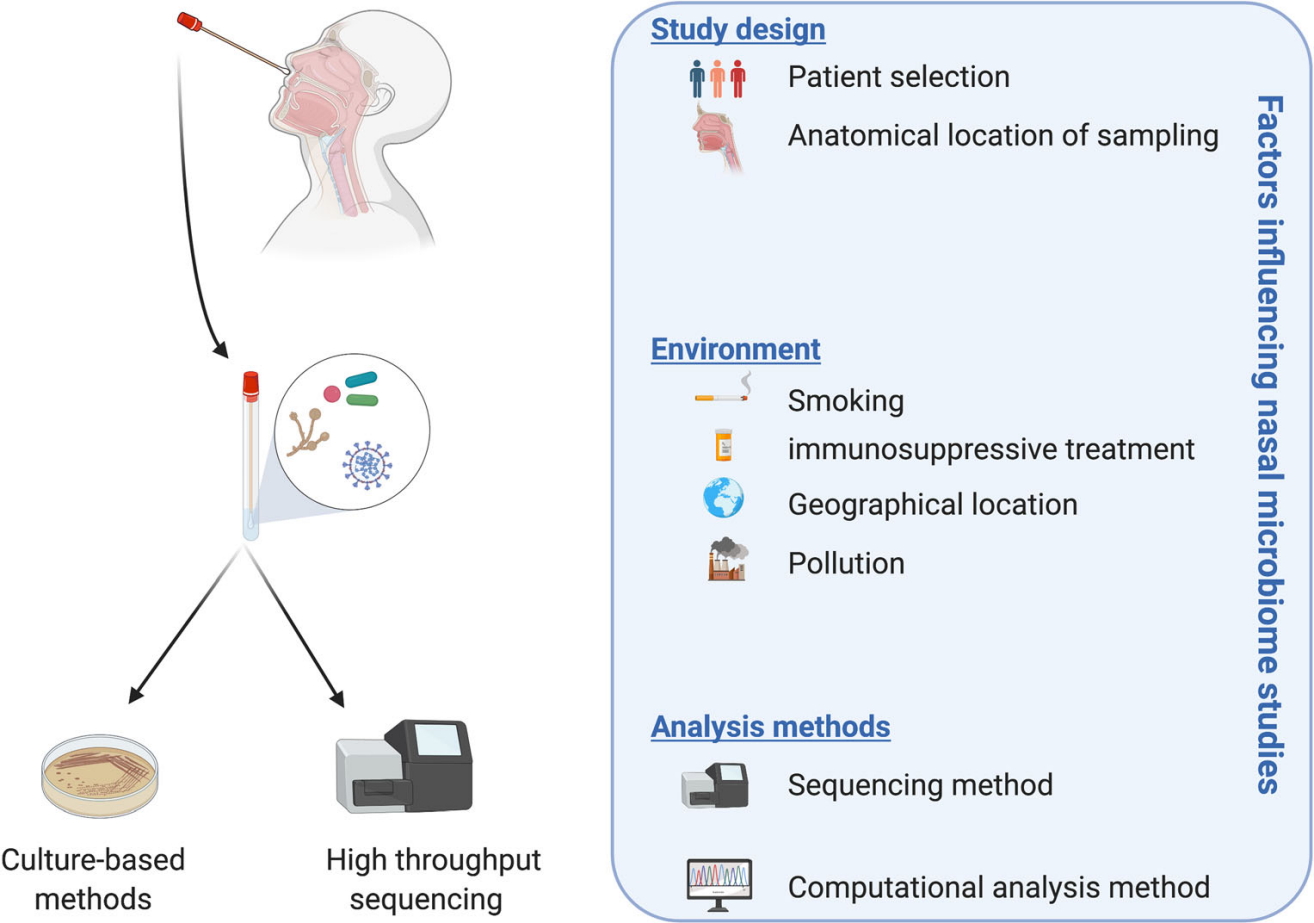
Overview of factors potentially influencing the composition of nasal microbial communities. Created with BioRender.com

Studies defining the microbiome in AAV in general, and GPA in particular, are still in its infancy. Whereas all nasal microbiome studies performed to date point to a state of dysbiosis, especially at the time of active disease, a direct comparison of the data is hampered due to differences in patient selection, treatment (antibiotic as well as steroid treatment), sampling locations, sequencing methods, and computational strategies for data analyses. In addition, it is well known that many environmental factors such as smoking, geographical location, and air pollution impact the composition of the nasal microbiota as well [44] (Fig. 2).

The reported microbiome data of the upper respiratory tract also reminds us that alterations in the composition of microbial communities and their interaction with the host are complex dynamic processes reaching far beyond colonization of *S. aureus* alone. Therefore, in future studies, approaches that combine descriptive microbiome studies with hypothesis-driven research will be essential to elucidate causative mechanisms that may be amenable for therapeutic intervention. Importantly, such studies should take into account the complex bi-directional relationship between the local and systemic host immune system and the colonizing microbial communities. For example, studies could be designed to include a detailed analysis of the nasal microbiome complemented with single-cell analyses of the nasal epithelium, proteomic profiling of nasal secretions, and characterization of systemic antimicrobial immune responses. Ideally, such studies should be performed in a longitudinal fashion and include patients from different geographical locations. Key to the success of these studies will be the standardization of patient selection, sample preparation and storage, as well as sequencing and computational analysis methods. Undoubtedly, this requires a multinational collaborative effort of multiple disciplines and the involvement of the entire vasculitis clinical research community.
